# Rational inhibitor design for *Pseudomonas aeruginosa* salicylate adenylation enzyme PchD

**DOI:** 10.1007/s00775-022-01941-8

**Published:** 2022-05-05

**Authors:** Catherine L. Shelton, Kathleen M. Meneely, Trey A. Ronnebaum, Annemarie S. Chilton, Andrew P. Riley, Thomas E. Prisinzano, Audrey L. Lamb

**Affiliations:** 1 Department of Molecular Biosciences, University of Kansas, Lawrence, KS 66045 USA; 2 Present Address: Department of Chemistry and Biochemistry, Northern Kentucky University, Highland Heights, Kentucky 41099 USA; 3 Present Address: Department of Chemistry, University of Texas San Antonio, San Antonio, TX 78249 USA; 4 Department of Chemistry, University of Kansas, Lawrence, KS 66045 USA; 5 Present Address: Roy and Diana Vagelos Laboratories, Department of Chemistry, University of Pennsylvania, Philadelphia, PA 19104-6323 USA; 6 Present Address: Department of Pharmaceutical Sciences, College of Pharmacy, University of Illinois at Chicago, Chicago, IL 60612 USA; 7 Department of Medicinal Chemistry, School of Pharmacy, University of Kansas, Lawrence, KS 66045 USA; 8 Present Address: Department of Pharmaceutical Sciences, College of Pharmacy, University of Kentucky, Lexington, KY 40536-0596 USA

**Keywords:** Adenylation domain, *Pseudomonas aeruginosa*, Inhibitor design, Antibiotic resistance, Pyochelin

## Abstract

*Pseudomonas aeruginosa* is an increasingly antibiotic-resistant pathogen that causes severe lung infections, burn wound infections, and diabetic foot infections. *P. aeruginosa* produces the siderophore pyochelin through the use of a non-ribosomal peptide synthetase (NRPS) biosynthetic pathway. Targeting members of siderophore NRPS proteins is one avenue currently under investigation for the development of new antibiotics against antibiotic-resistant organisms. Here, the crystal structure of the pyochelin adenylation domain PchD is reported. The structure was solved to 2.11 Å when co-crystallized with the adenylation inhibitor 5′-*O*-(N-salicylsulfamoyl)adenosine (salicyl-AMS) and to 1.69 Å with a modified version of salicyl-AMS designed to target an active site cysteine (4-cyano-salicyl-AMS). In the structures, PchD adopts the adenylation conformation, similar to that reported for AB3403 from *Acinetobacter baumannii*.

**Graphical abstract:**

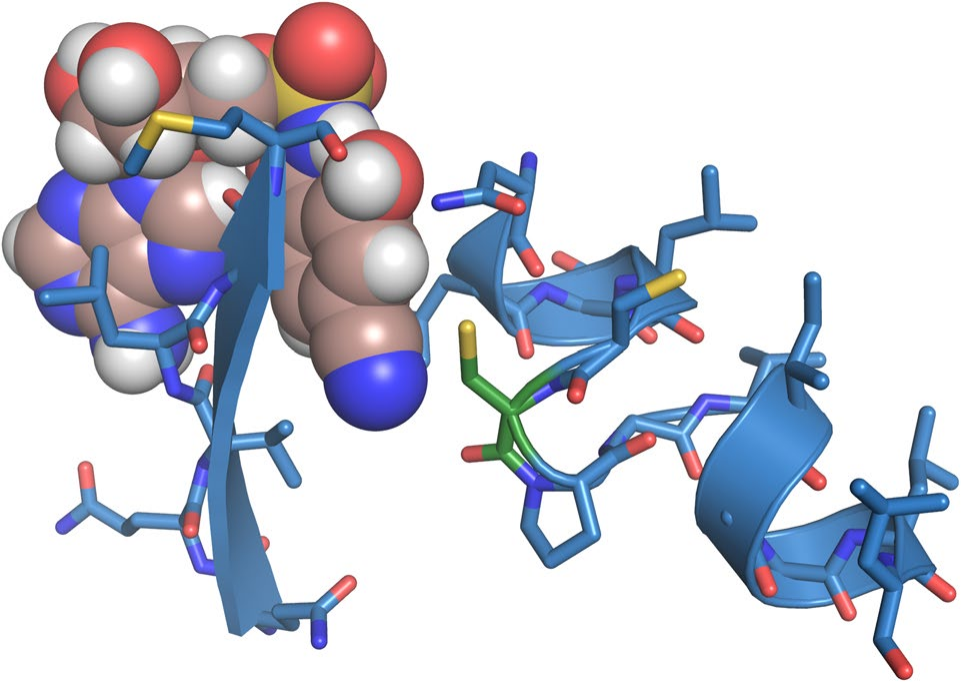

## Introduction

Pathogenic organisms compete with their biological hosts for metal nutrients, particularly iron. The host limits free metals in an effort to starve bacteria of needed metals, a phenomenon termed nutritional immunity [1]. To circumvent nutritional immunity, bacteria have developed metal acquisition systems that include synthesis and secretion of metal chelators that scavenge metal from the host and are then imported back into the bacteria. Pyochelin is a metallophore produced by the antibiotic-resistant pathogen *Pseudomonas aeruginosa*, and while capable of binding a variety of metals, pyochelin’s highest affinity is for Fe^3+^ [2, 3]. Highlighting the nutritional importance of pyochelin-mediated iron uptake are early publications demonstrating that pyochelin stimulates bacterial growth in murine infections [4] and that pyochelin is needed for *P. aeruginosa* virulence [5].

Like many natural products, the metallophore pyochelin is synthesized by a non-ribosomal peptide synthase (NRPS). NRPS modules are multidomain proteins (adenylation domain, peptidyl carrier domain, condensation domain) that construct small peptides in an assembly-line fashion. The adenylation domain activates each new amino acid prior to its integration into the growing peptide chain by catalyzing the formation of an aminoacyl-AMP. Following activation, the peptidyl carrier domain transfers the activated product to the condensation domain where it is joined to the growing peptide chain. This process is repeated with additional NRPS modules until the full molecule is assembled. Pyochelin is assembled from three precursor molecules: one salicylate and two cysteines. The biosynthesis is achieved by three NRPS modules, two accessory enzymes, and one stand-alone tailoring enzyme [6]. PchD is a stand-alone adenylation enzyme and is part of the initiation module of pyochelin biosynthesis.

**Fig. 1:**
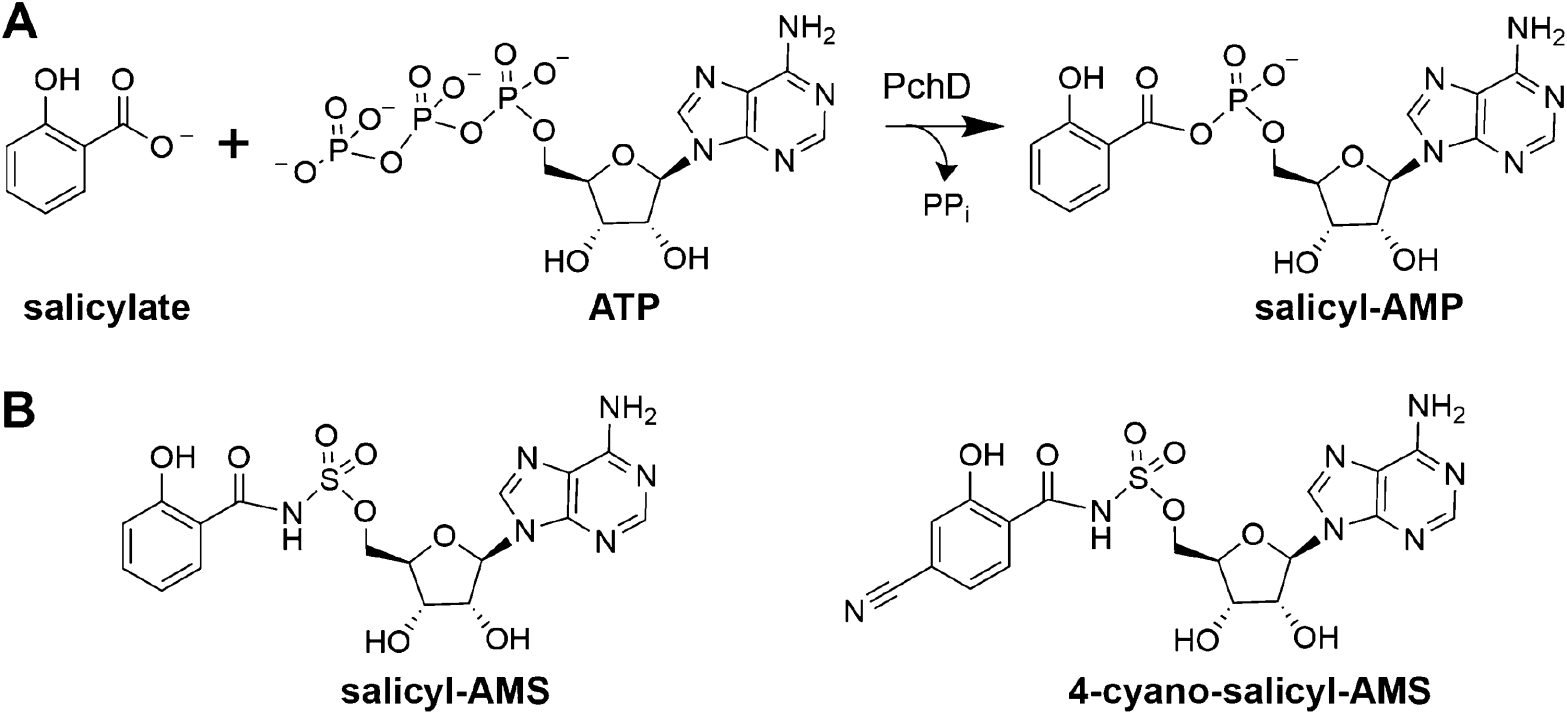
**A** PchD catalyzes the adenylation of salicylate as the first step in pyochelin biosynthesis. **B** Salicyl-AMS, originally described by Ferreras et al. as an inhibitor of siderophore producing NRPS adenylation domains in *M. tuberculosis* and *Y. pestis* has been modified by the addition of a cyano group at C4 of the salicylate ring to produce 4-cyano-salicyl-AMS

The role of PchD is twofold. In the adenylation reaction, salicyl-AMP is formed from salicylate and ATP, releasing pyrophosphate (Fig. 1A). Next, the thiol of the phosphopantetheinyl (Ppant) prosthetic group of the *N*-terminal peptidyl carrier protein domain of PchE performs a nucleophilic attack on the carboxyl ester of salicyl-AMP, releasing AMP and transferring the salicyl moiety to the Ppant tether, and priming the first module in the biosynthetic pathway. A homolog to PchD is also found in the major pathogen *Mycobacterium tuberculosis* (MbtA) which acts as a similar salicylate-AMP ligase in the biosynthetic pathway for the siderophore mycobactin [7]. Both PchD and MbtA are part of the family of adenylating enzymes that, in addition to including adenylation domains from NRPS modules, also encompasses firefly luciferase and acyl and aryl-CoA synthetases and is, therefore, termed the ANL superfamily (**A**cyl-CoA synthetases, **N**RPS adenylation domains, and **L**uciferase enzymes) [8]. Previously solved structures have captured other NRPS adenylation domains in either the adenylation conformation or in the thioester-forming conformation [9]. These structures serve as a template for identifying the catalytic relevance of the structures described here and of other adenylation domain structures.

*P. aeruginosa* and *M. tuberculosis* have been listed by the Centers for Disease Control and Prevention as serious threats due to their increasing antibiotic resistance [10], which led to the hypothesis that NRPS adenylation domains such as PchD and MbtA are attractive targets for the development of new antibiotics [11]. Noting that mechanistically related adenylation domains (*i.e.* aminoacyl tRNA-synthetases) were inhibited by analogues of their acyl-AMP intermediates, Finking et al. first described the use of sulfonyladenosine (AMS) inhibitors for NRPS adenylation domains [12]. Ferreras et al. subsequently pioneered the development of inhibitors against the NRPS adenylation domains in *M. tuberculosis* (MbtA), *Yersinia pestis* (YbtE; *Y. pestis* is the causative agent of plague), and *Pseudomonas aeruginosa* (PchD) [13]. They synthesized and tested the inhibitor 5′-*O*-(N-salicylsulfamoyl)adenosine (salicyl-AMS) (Fig. 1B) and demonstrated nanomolar K_i_ values for MbtA, YbtE and PchD and micromolar IC_50_ values for growth inhibition of *M. tuberculosis* and *Y. pestis*. Since that time, salicyl-AMS has been the focus of multiple structure–activity studies [14–23]. The work presented here describes a modification to salicyl-AMS that exploits the presence of an active site cysteine residue near the salicyl ring of the inhibitor. We hypothesized that the extension of an electrophile off the salicyl ring would provide the opportunity for covalent binding between the cysteine in the active site and the newly introduced electrophile. Using a method based on the one developed by Ferreras [13], a nitrile group was introduced on C4 of the aryl group to produce 4-cyano-salicyl-AMS (Fig. 1B).

The apo-structure of the adenylation domain MbtA from *Mycobacterium smegmatis*, which shares 69.2% sequence identity with *M. tuberculosis* MbtA, has been determined previously [24]. The structures presented here represent the *P. aeruginosa* adenylation domain PchD with either salicyl-AMS or 4-cyano-salicyl-AMS. In contrast to the MbtA structure which was solved in an apparent non-catalytic conformation, the PchD structures presented here demonstrate the catalytically relevant adenylation conformation as well as important active site contacts with the ligands.

## Methods

### Cloning, expression, and purification of *pchD*

The *pchD* gene was amplified from *Pseudomonas aeruginosa* PAO1 genomic DNA by polymerase chain reaction by use of Herculase polymerase (Agilent) supplemented with 6–10% DMSO and 3% glycerol. The forward primer (5′-TAT ATT CAT ATG ACT TCC TCG CCC GTC ACC-3′) includes an *Nde*I site (underlined), whereas the reverse primer (5′-TTA TAT GGA TCC TCA TGC GCG GGC CTC CAG-3′) contains a *BamH*I site (underlined). The amplified 1665 basepair fragment was digested with *Nde*I and *BamH*I and ligated into the pET28b plasmid (Novagen) digested with the same enzymes. The resulting plasmid encodes the gene for the PchD protein with an N-terminal histidine tag. BL21(DE3) *pLysS E. coli* (Invitrogen) containing the PchD expression plasmid were grown in LB broth containing 50 μg/mL kanamycin at 37 °C with shaking (225 rpm). When OD_600_ was ~0.7, the temperature was reduced to 30 °C and protein expression was induced with the addition of isopropyl β-d-thiogalactopyranoside to a final concentration of 200 μM. The cells were harvested by centrifugation (6000*×g*, 10 min, 4 °C) after ~4 h. The cell pellet was resuspended in 5 mL of 25 mM Tris–HCl pH 8, 500 mM NaCl per 2 L of culture. Cells were disrupted by use of a French pressure cell (35,000 psi), and cellular debris was removed by centrifugation (12,000*×g*, 1 h, 4 °C). The supernatant was applied to a chelating Sepharose fast-flow column (Amersham Biosciences) charged with nickel chloride and pre-equilibrated in 25 mM Tris–HCl pH 8, 500 mM NaCl, 50 mM imidazole (buffer A). PchD protein eluted at 100 mM imidazole in a linear gradient of 50–300 mM in buffer A. The pooled fractions were applied to a Superdex 200 size-exclusion column (Amersham Biosciences) equilibrated with 25 mM Tris–HCl pH 8, 150 mM NaCl, 10% glycerol, 2 mM DTT. The fractions containing PchD were pooled and concentrated by use of an Amicon stirred cell with a YM-10 membrane to 17 mg/mL as determined by the Bradford assay and stored at −80 °C.

### Synthesis of salicyl-AMS and 4-cyano-salicyl-AMS

All chemical reagents were purchased from commercial suppliers and used without further purification. Flash column chromatography was performed on silica gel (40–63 mm) from Sorbent Technologies. Separation was performed with a Teledyne Isco CombiFlash Rf.

5′-*O*-(N-salicylsulfamoyl)adenosine (salicyl-AMS). The synthesis of salicyl-AMS was performed as previously described in Ferreras et al. [13]. The spectroscopic data of the purified product agreed with literature values.

((2R,3S,4R,5R)-5-(6-amino-9HH-purine-9-yl)-3,4-dihydroxytetrahydrofuran-2-yl)methyl (4-cyano-2-hydroxybenzoyl)sulfamate (4-cyano-salicyl-AMS). The synthesis of 4-cyano-salicyl-AMS was also performed as previously described in Ferreras et al., except the starting material differed by using 4-cyano-2-hydroxybenzoic acid (ArkPharma) instead of 2-hydroxybenzoic acid. Briefly, a solution of 4-cyano-2-hydroxybenzoic acid (39 mg, 0.24 mmol) and 1,1-carbonyldiimidazole (93 mg, 0.575 mmol) in anhydrous acetonitrile (5 mL) were stirred under argon atmosphere at 60 °C for 2 h. A mixture of 1,8-diazabicyclo[5.4.0]undec-7-ene (36 µL, 0.24 mmol) and ((2*R*,3*R*,4*R*,5*R*)-5-(6-amino-9*H*-purin-9-yl)-3,4-bis((*tert*-butyldimethylsilyl)oxy)tetrahydrofuran-2-yl)methyl sulfamate (92 mg, 0.16 mmol) in 1 mL of anhydrous acetonitrile were added to the reaction dropwise and the reaction stirred at 60° C for an additional 30 min. The reaction was then diluted with H_2_O and extracted with ethyl acetate (4 × 20 mL). The organic layer was washed with HCl (1 M, 1 × 20 mL), NaHCO_3_ (sat’d, 1 × 20 mL), and brine (1 × 20 mL). After being dried with MgSO_4_, the organic phase was decanted and concentrated under reduced pressure and the TBS-protected product was purified using flash chromatography (0–30% MeOH gradient in CH_2_Cl_2_ over 40 min). Fractions containing the TBS-protected product were collected and concentrated *in vacuo* and left under high vacuum overnight, yielding a red–orange solid (25 mg). The resulting solid was redissolved in THF (1.6 mL) and was treated dropwise with a 1 M solution of tetrabutylammonium fluoride. The reaction was stirred at room temperature for 30 min before being concentrated under reduced pressure. The product was isolated by flash chromatography (0–35% MeOH gradient in CH_2_Cl_2_) to yield a red–orange solid (13 mg, 16%). The product was 59% pure based on the UV–VIS trace from LC-HRMS. **HRMS:** [M + H] + 492.0859 (calcd), 492.0940 (found).

### Crystallization

PchD was co-crystallized with salicyl-AMS at 25 °C using the hanging drop method. Before crystallization, PchD (340 µM) was incubated with 3.4 mM (10×) salicyl-AMS, respectively, on ice for 30 min. The drop was made by mixing 1.5 µl protein solution with 1.5 µl well solution consisting of 20% PEG 8000, 0.2 M ammonium acetate, 0.1 M MES pH 5.4, 0.03 M ammonium chloride. Crystals grew to about 0.3 × 0.025 × 0.01 mm in 1 week. For data collection, crystals were transferred to reservoir solution containing 20% (v/v) ethylene glycol as a cryoprotectant and flash cooled at −160 °C.

PchD was co-crystallized with 4-cyano-salicyl-AMS at 25 °C using the hanging drop method. Prior to crystallization, 4-cyano-salicyl-AMS was added to the 228 µM PchD stock, to a final concentration of 1000 µM (~fourfold excess), and was incubated on ice for 30 min. The drop was made by mixing 1.5 µl protein solution with 1.5 µl well solution consisting of 26% PEG 8000, 0.2 M ammonium acetate, 0.1 M MES pH 5.6, 0.03 M ammonium chloride. In the presence of 4-cyano-salicyl-AMS crystals grew to about 0.3 × 0.025 × 0.01 mm in one week. Crystals did not grow in the absence of the inhibitor. For data collection, crystals were transferred to reservoir solution containing 20% (v/v) ethylene glycol as a cryoprotectant and flash cooled at −160 °C.

### Data collection and processing

PchD salicyl-AMS co-crystal diffraction data (1° oscillation images for a total of 270°) were collected at the Stanford Synchrotron Radiation Laboratory (Stanford, CA) beamline 7–1 with a wavelength of 1.1271 Å at 100 K. The exposure time per frame was 60 s with an attenuation of 0% and a crystal to detector distance of 200 mm. The data were indexed and scaled with XDS to 2.11 Å [25]. The crystals were assigned to the space group C2 with unit cell dimensions *a* = 177.06 Å, *b* = 44.83 Å, *c* = 67.20 Å, and *β* = 99.09°.

PchD 4-cyano-salicyl-AMS co-crystal diffraction data (0.15° oscillation images for a total of 163.05°) were collected at the Stanford Synchrotron Radiation Laboratory (Stanford, CA) beamline 12–2 with a wavelength of 0.9795 Å at 100 K. The exposure time per frame was 0.2 s with an attenuation of 57.5% and a crystal to detector distance of 230.8 mm. The data were indexed and scaled with XDS to 1.69 Å [25]. The crystals were assigned to the space group C2 with unit cell dimensions *a* = 177.06 Å, *b* = 44.85 Å, *c* = 66.61 Å, and *β* = 99.18°.

### Structure solution and refinement

Molecular replacement calculations were performed using Phaser [26] in the PHENIX [27] program suite. The model used for this structure was dihydroxybenzoate adenylation domain (DhbE) from *Bacillus subtilis* (PDB 1MDB) with waters and ligand removed [28]. For the salicyl-AMS structure molecular replacement calculations yielded a clear solution with a log likelihood gain of 624.78 and a TFZ score of 16.7. Model building and refinement were performed using Coot [29] and Phenix refine [30] and waters were placed by Phenix refine, corrected manually and verified using a 2*mF*_*o*_*–F*_*c*_ electron-density map contoured at 1.5σ following a round of refinement. The salicyl-AMS was generated in eLBOW [31] and placed using LigandFit [32, 33]. The final model includes one molecule with residues 14–201 and 206–540, one salicyl-AMS molecule, and 139 water molecules. Geometry analysis was performed by *MolProbity *[34]. The salicyl-AMS structure was used as the molecular replacement model for the 4-cyano-salicyl-AMS structure and the molecular replacement calculations yielded a clear solution with a log likelihood gain of 21,293 and a TFZ score of 116.8. Model building and refinement were performed using Coot [29] and Phenix refine [30]. The 4-cyano-salicyl-AMS was generated in eLBOW [32] and placed using LigandFit [32, 33]. Waters were placed by Phenix refine and validated manually with each round of refinement. The final model includes one molecule with residues 13–542, one 4-cyanosalicyl-AMS molecule, and 292 water molecules. A comparison of structures and calculation of RMSD values were performed using PDBeFold [35]. Structure figures were generated in PyMOL (PyMOL Molecular Graphics System, version 2.0, Schrödinger, LLC).

## Results and discussion

**Fig. 2:**
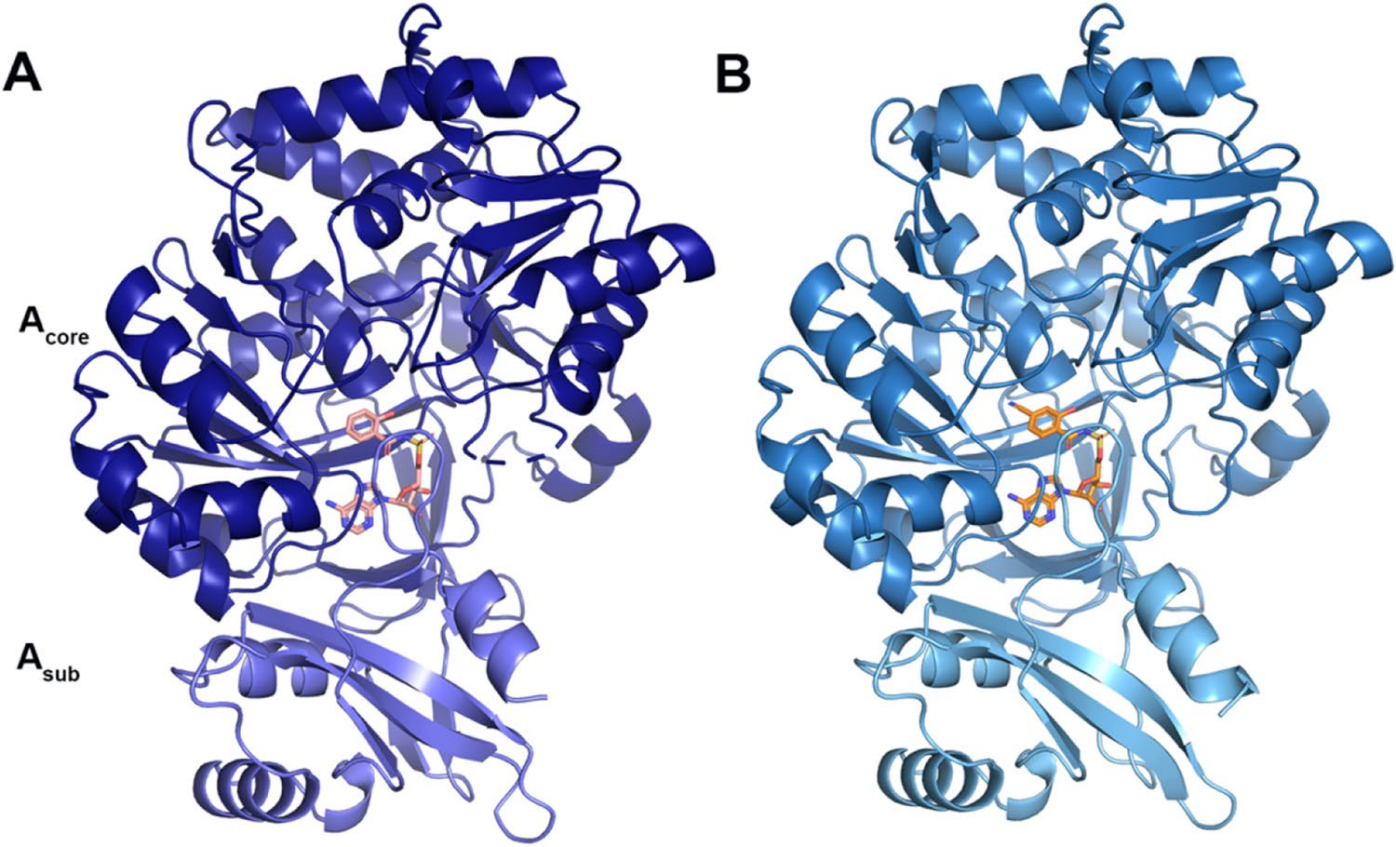
**A** PchD bound to salicyl-AMS (salmon) shown in midnight blue. **B** PchD bound to 4-cyanosalicylAMS (orange) shown in skyblue

Previous attempts to crystalize the N-terminally his-tagged PchD in the absence of a ligand were unsuccessful [36] but crystallization was easily achieved when either salicyl-AMS or 4-cyano-salicyl-AMS were present. Additional confirmation that the correct ligands were produced and co-crystalized was demonstrated retrospectively by the high-resolution structures, despite sub-optimal purity for the 4-cyano-salicyl-AMS synthesis. The co-crystal of PchD and salicyl-AMS diffracted to 2.11 Å resolution and X-ray diffraction data were collected on Stanford Synchrotron Radiation Lightsource beamline 7-1 (Fig. 2A; Table 1). The co-crystal of PchD and 4-cyano-salicyl-AMS diffracted to 1.69 Å resolution and X-ray diffraction data were collected on Stanford Synchrotron Radiation Lightsource beamline 12-2 (Fig. 2B; Table 1). Molecular replacement was performed using the dihydroxybenzoate adenylation domain (DhbE) from *Bacillus subtilis* (PDB 1MDB). PchD is comprised of a large N-terminal subdomain (*A*_core_) consisting of residues 1–439 and a smaller C-terminal subdomain (*A*_sub_) consisting of residues 441–542 with residue K440 designating the hinge location that allows a range of motion between the two domains (Fig. 2). The *A*_core_ subdomain is made of two αβαβα regions that form a sandwich and a β-barrel. The *A*_sub_ of PchD is comprised of a central three stranded β-sheets surrounded by three helices. Regardless of the ligand bound, the two structures are in identical conformation with an rmsd of 0.15 Å over 523 Cα.

**Table 1:** Data collection and refinement statistics for PchD structures

	PchD salicyl-AMS PDB: 7TYB	PchD 4-cyano-salicyl-AMS PDB: 7TZ4
**Data collection**^a^		
Beamline	7-1	12-2
Wavelength (Å)	1.1271	0.9795
Space group	C2	C2
Cell dimensions; *a*, *b*, *c* (Å), *β* (°)	177.06, 44.83, 67.20, 99.09	177.06, 44.85, 66.61, 99.18
Resolution (Å)	39.48–2.11 (2.18–2.11)	36.93–1.69 (1.72–1.69)
* R*_merge_^b^	0.182 (0.828)	0.083 (0.671)
* R*_pim_	0.091 (0.426)	0.062 (0.526)
Total observations	165,590 (11,422)	174,245 (6247)
Total unique observations	29,938 (2235)	56,335 (2309)
Mean ((*I*)/sd(*I*))	7.5 (2.2)	8.6 (2.0)
Completeness (%)	99.1 (90.5)	97.3 (77.0)
Redundancy	5.5 (5.1)	3.1 (2.7)
Wilson B-factor	17.06	13.56
**Refinement**		
Resolution (Å)	37.01–2.11 (2.17–2.11)	36.93–1.69 (1.74–1.69)
* R*_cryst_^c^	0.1677 (0.2317)	0.1487 (0.2255)
* R*_free_	0.2267 (0.3190)	0.1773 (0.2699)
Total unique observations	27,952 (1680)	56,238 (3347)
No. of non-hydrogen atoms	4245	4439
Protein	4074	4113
Ligand	32	34
Water	139	292
rms deviation bonds (Å)	0.017	0.009
rms deviation angles (°)	1.65	1.070
Overall mean B-factor (Å^2^)	23.99	17.70
**Ramachandran plot analysis**^d^		
Favored region	98.27	98.50
Allowed region	1.54	1.50
Outlier region	0.19	0.00

^a^Data indexed and scaled with XDS

^b^*R*_merge_ = Σ_*h*_|*I*_*h*_—< *I* >|/*Σ*_*h*_*I*_*h*_, where *I*_h_ is the intensity of reflection *h*, and < *I* > is the mean intensity of all symmetry-related reflections

^c^*R*_cryst_ = *Σ*||*F*_*o*_|—|*F*_*c*_||/*Σ*|*F*_*o*_|, *F*_*o*_ and *F*_*c*_ are observed and calculated structure factor amplitudes. Five percent of the reflections were reserved for the calculation of *R*_free_

^d^Calculated with Molprobity

**Fig. 3:**
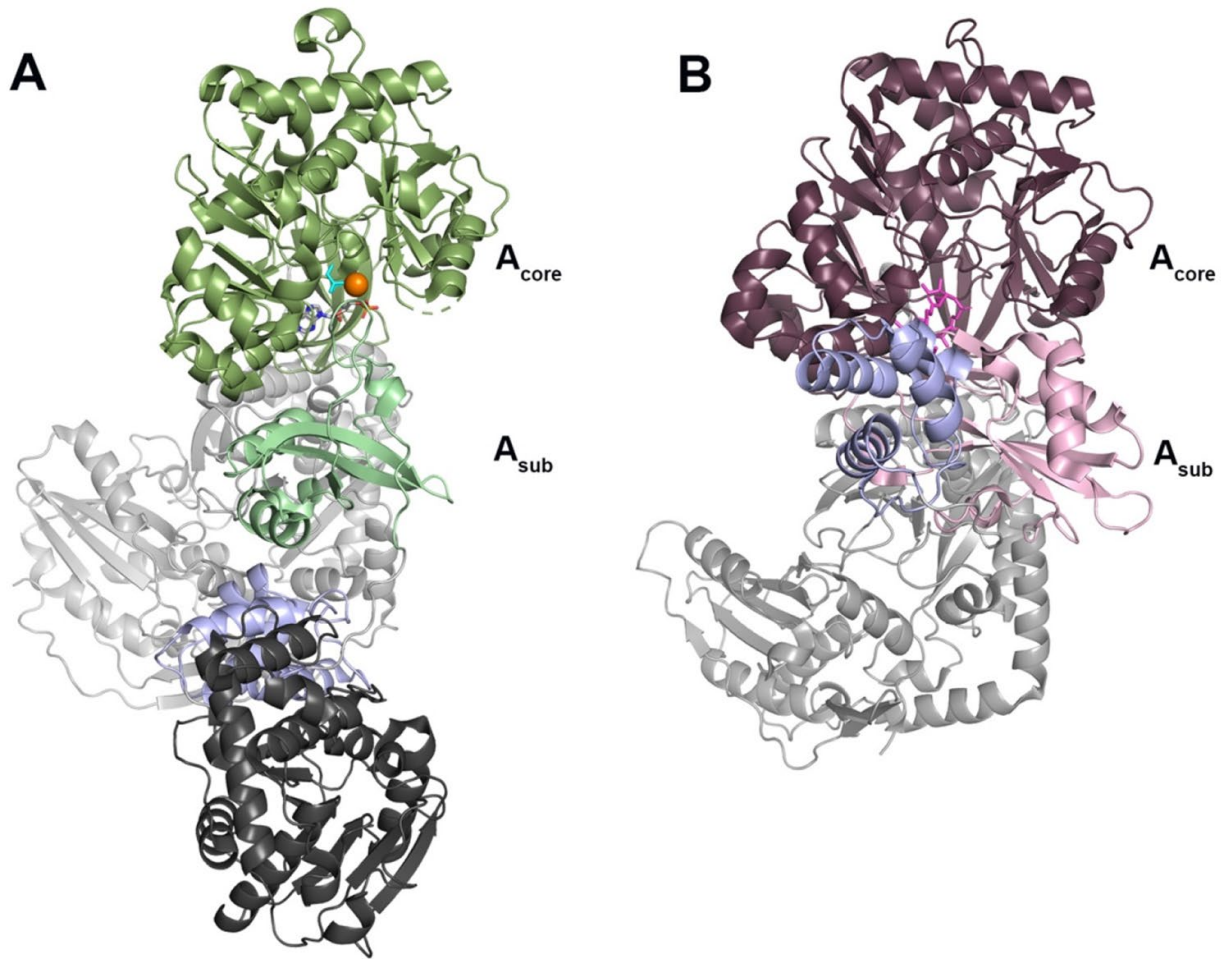
**A** The adenylation conformation represented by the full NRPS module from AB3403 (4ZXI) with the adenylation domain shown in green, the condensation domain shown in light grey, the PCP domain shown in light purple, and the thioesterase domain shown in dark grey. Ligands shown in the adenylation domain include AMP (grey), Mg2^+^ (orange) and glycine (cyan) (**B**). The thioester-forming conformation represented by the EntF NRPS module (5T3D) with the adenylation domain shown in plum/pink, the condensation domain shown in light grey, and the PCP domain shown in light purple. The mechanism-based inhibitor Ser-AVS is shown in fuchsia

Drake et al. crystallized two complete NRPS modules, each trapped in a different stage of the catalytic cycle [9]. The adenylation domains of these two modules provide useful comparisons for determining the catalytic conformation of PchD. The NRPS module from *Acinetobacter baumannii*, implicated in biofilm formation, has been termed AB3403. AB3403 was co-crystalized with Mg·ATP and glycine and demonstrates the adenylation conformation shown in Fig. 3A (PDB 4ZXI) [9]. The EntF NRPS module from *Escherichia coli* for the production of the siderophore enterobactin was co-crystalized with a mechanism-based inhibitor to capture the thioester-forming conformation and is shown in Fig. 3B (PDB 5T3D) [9].

**Fig. 4:**
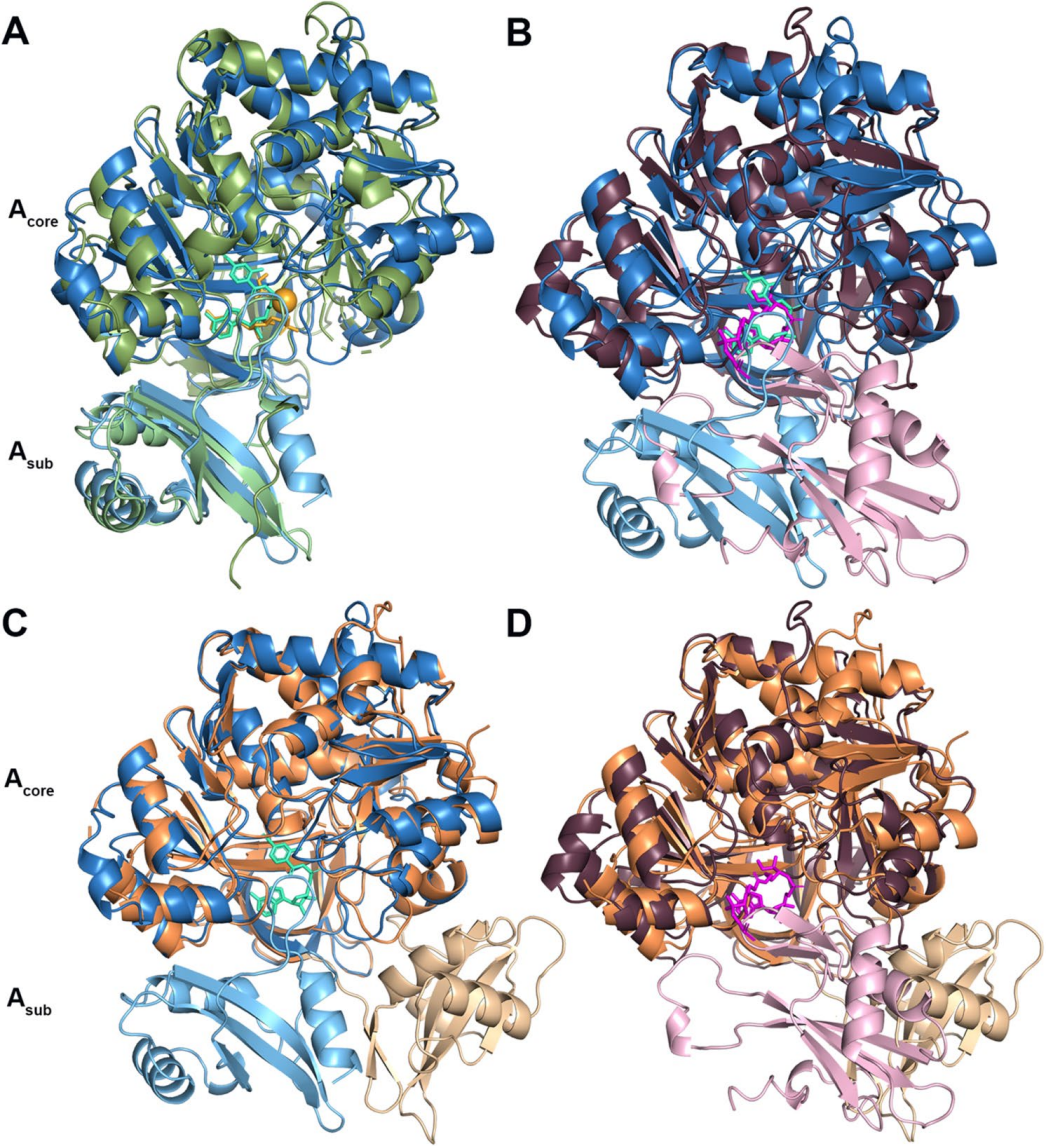
**A** PchD (blue) closely aligns with the adenylating conformation demonstrated by the AB3403 adenylation domain (green). 4-cyano-salicyl-AMS ligand of PchD shown in teal. **B** The EntF adenylation domain (*A*_core_:plum, *A*_sub_: pink) demonstrates the thioester-forming conformation and was co-crystalized with the ligand serine adenosine vinylsulfonamide (magenta). The *A*_sub_ of MbtA (wheat; *A*_core_: orange) does not align with either the **C** adenylation or the **D** thioester conformation

Visualization of these two states provided confirmation that the motion of the A_sub_ adenylation domain is important for catalysis [8, 9]. PchD overlays very closely with the adenylate-forming conformation of the AB3403 adenylation domain with an rmsd of 2.03 Å over 438 Cα (Fig. 4A). However, when the same comparison is made to the thioester-forming conformation of the EntF adenylation domain, the rmsd is similar (2.10 Å) but for only 364 Cα (Fig. 4B). The large difference in α-carbons for the comparison is due to the rotation in the *A*_sub_ between the adenylation-forming and thioester-forming conformations. Separate alignments of the *A*_core_ and *A*_sub_ provide a more complete picture. Comparing these two regions for PchD and AB3403, the *A*_core_ has an rmsd of 2.10 Å over 344 Cα while the *A*_sub_ has an rmsd of 1.49 Å over 93 Cα. When the same regions are aligned between PchD and EntF the *A*_core_ has an rmsd of 2.05 Å over 356 Cα and the *A*_sub_ has an rmsd of 1.86 Å over 89 Cα. The presence of either the salicyl-AMS ligand or the 4-cyano-salicyl-AMS ligand was an absolute requirement for crystallization of PchD, likely because it locks down the highly mobile *A*_sub_ subdomain into the adenylate-forming conformation.

The structure of a homologous free-standing adenylation enzyme MbtA from *M. smegmatis* was published by Vergnolle et al. [24]. Figure 4C shows an overlay of MbtA and PchD demonstrating remarkable similarity of the two enzymes except in the conformation of the *A*_sub_ (rmsd 1.07 Å for 409 Cα). Because both PchD and MbtA use salicylate as a substrate, it is unsurprising that they are so similar. This similarity is further evident by separate comparisons of the *A*_core_ and *A*_sub_ regions because this eliminates the differences in orientation of the *A*_sub_. For the *A*_core_ there is an rmsd of 1.05 Å for 406 Cα and for the *A*_sub_ the rmsd is 1.30 Å for 96 Cα. The conformation of the MbtA *A*_sub_ does not conform to either the adenylation conformation (Fig. 4C) or the thioester conformation (Fig. 4D). Because the MbtA structure was crystallized without a ligand, it is possible that the orientation of the *A*_sub_ maximized crystal contacts making the conformation an artefact of crystallography.

**Fig. 5:**
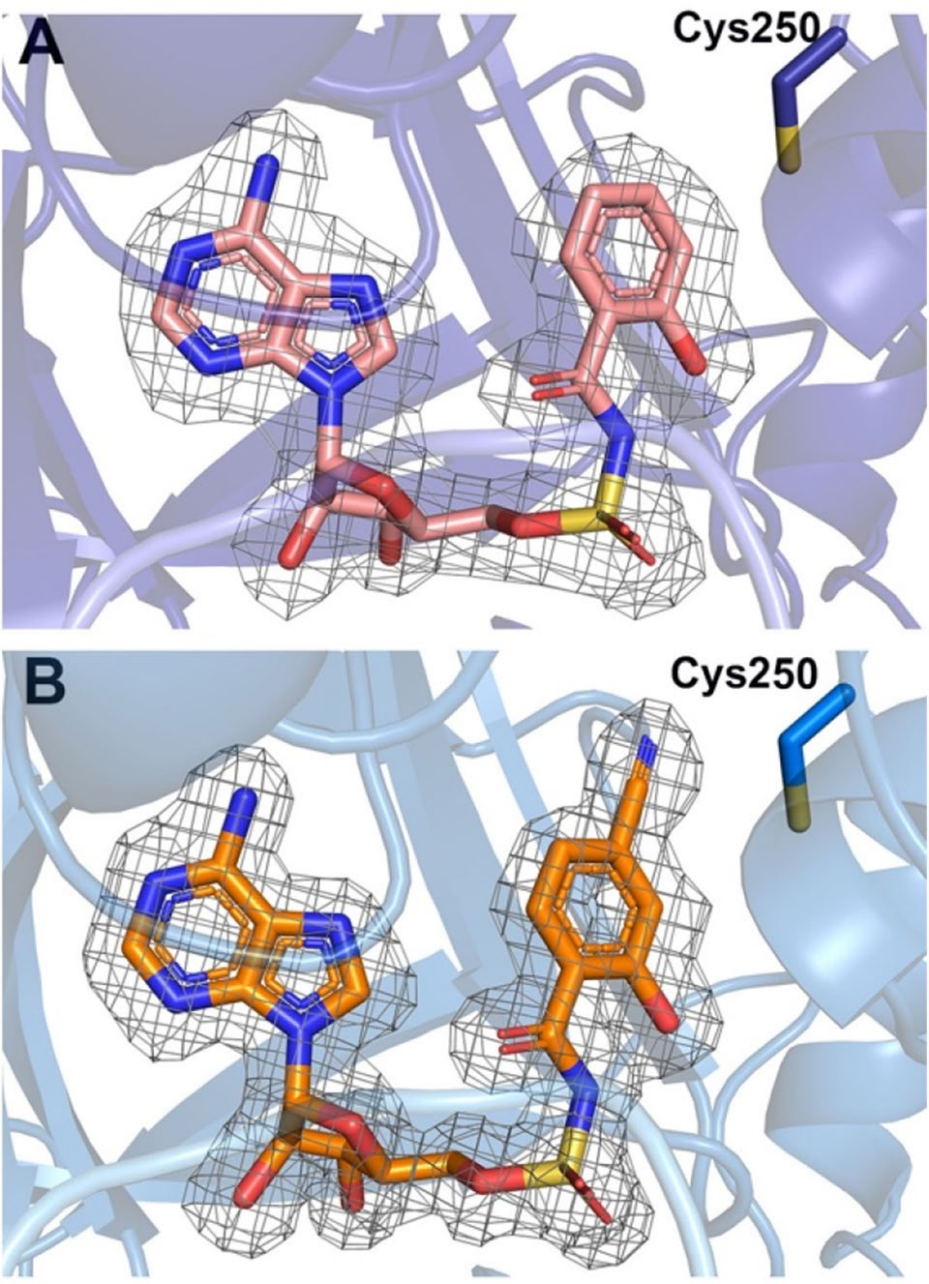
**A** Relative position of Cys250 to the salicyl-AMS inhibitor. **B** Relative position of the 4-cyano-salicyl-AMS inhibitor. Inhibitor densities are shown with a polder map drawn at 3σ

Analysis of the salicyl-AMS-bound PchD active site revealed that Cys250 is located near to the salicylate ring of salicyl-AMS (Fig. 5). Therefore, a salicyl-AMS inhibitor derivative was designed and synthesized with a cyano group at C4 that could extend towards Cys250 with the expectation of covalent bond formation by the electrophilic warhead would convert the potent inhibitor into a targeted covalent inhibitor [37]. As anticipated, the nitrile group of 4-cyano-salicyl-AMS and Cys250 were in close proximity (3.8 Å). The presence and location of the cyano group were verified by calculating a polder map (Fig. 5B). Surprisingly, while a slight change in the orientation of the Cys250 side chain might allow for bond formation, the covalent bond was not observed in the crystal structure. The absence of bond formation may indicate the need for a change in the orientation of the cyano group relative to Cys250. Alternatively, the active site may alter the pKa of the cysteine such that deprotonation for bond formation is not favored. An increase in the pH of the crystallization conditions may facilitate bond formation.

**Fig. 6:**
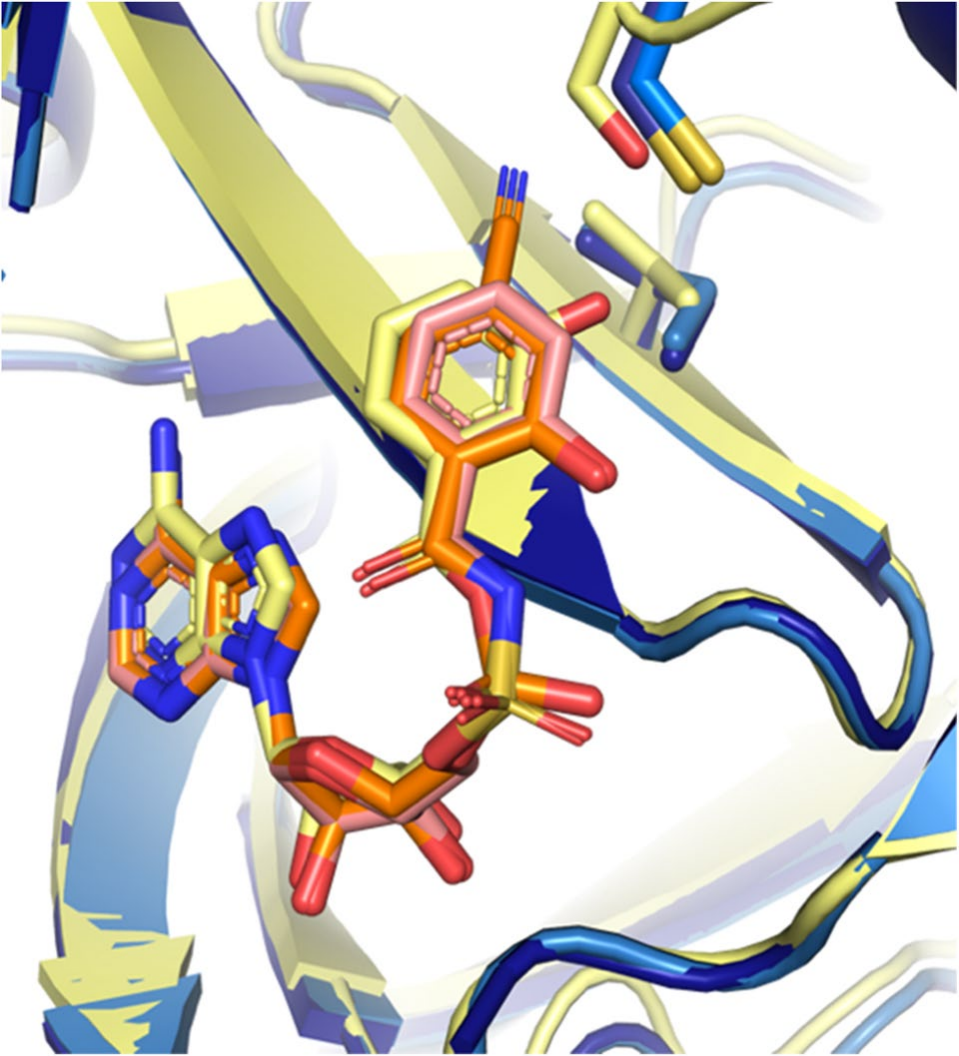
DhbE adenylation domain bound to DHB-adenylate (1MDB) shown in yellow overlayed with both salicyl-AMS PchD (salmon inhibitor, midnight blue structure) and 4-cyano-salicyl-AMS bound PchD (orange inhibitor, skyblue structure). Residues conferring specificity for salicylate are Cys250 and Ile347. For the DhbE structure these positions are a conserved serine and a valine which are hypothesized to more readily accommodate the second hydroxyl of DHB

DhbE is a free-standing adenylation domain in the biosynthetic pathway for the bacillibactin siderophore in *Bacillus subtilis*. The substrate for DhbE is 2,3-dihydroxybenzoic acid (DHB) which differs from salicylate only in the presence of a second hydroxyl group at the C3 position. The structure of DhbE bound to its adenylated product DHB-AMP was solved by May et al. and served as the molecular replacement search model for both the PchD structures reported here and for the MbtA structure [28]. The amino acid composition of the active site for DHB adenylation domains and salicylate adenylation domains is remarkably similar. In trying to identify which active site residues confer substrate specificity, May et al. hypothesized that two positions distinguish between DHB and salicylate. They noted that the Cys250 position (described above) was conserved in salicylate adenylation domains but that in DHB adenylation domains this same position is a serine which is needed to make room for the additional hydroxyl group on DHB. The second position is Ile347 in PchD (Leu250 in MbtA). In DhbE this position is a valine, again, to accommodate the second hydroxyl functional group (Fig. 6).

**Fig. 7:**
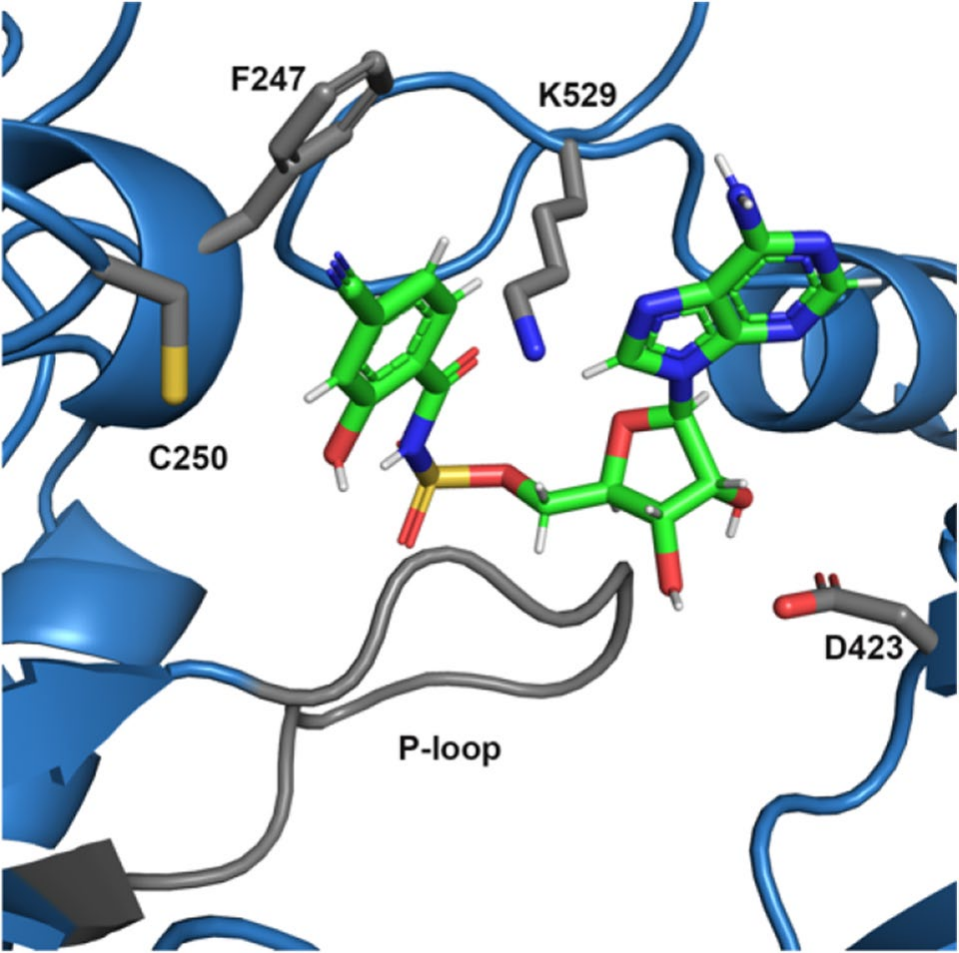
Contacts between active site residues and the inhibitor

The finding that PchD would not crystallize except in the presence of an inhibitor compound indicates that important contacts are formed between the compound and active site residues and serve to lock the enzyme in the adenylating conformation (Fig. 7). Lysine 529 of the *A*_sub_ is within 3 Å of both the sulfamate oxygen and the sugar oxygen and likely helps coordinate this region of the compound. Phenylalanine 247 likely forms a hydrophobic region for the salicylate ring, but is too distant (4.3–4.9 Å) to form pi–pi stacking interactions. The P-loop region, the phosphate-binding motif in ATP-binding enzymes, encompasses residues 200–210 (SGGTTGTPKLI) [28]. Though important for binding the native substrate (ATP), the P-loop appears to have little involvement in coordinating the 4-cyano-salicyl-AMS inhibitor. The P-loop remained quite mobile based on elevated B-factors in this region and broken density in the map. In the salicyl-AMS structure, the P-loop is even more disordered with insufficient density to fully build the loop.

## Conclusions

Using the potent salicyl-AMS inhibitor originally designed by Ferreras et al. [13], the structure of the stand-alone salicylate adenylation domain from *Pseudomonas aeruginosa* was determined to 2.11 Å. This is the first structure of an adenylation domain bound to the salicyl-AMS inhibitor and serves as an important visual model for making future modifications to continue to develop salicyl-AMS as a therapeutic drug. Indeed, the initial structure led to the design and synthesis of a proposed targeted covalent inhibitor, 4-cyano-salicyl-AMS, and a subsequent structure was determined to 1.69 Å. However, the novel inhibitor did not react with the active site cysteine as anticipated. Importantly, both structures were trapped in the catalytically relevant adenylation conformation due to the presence of the inhibitor. This work furthers the continuing story of targeting the adenylation domains of non-ribosomal peptide synthases for antimicrobial drug discovery, with an eye toward therapeutic intervention for infections caused by *Pseudomonas aeruginosa, Mycobacterium tuberculosis,* and *Yersinia pestis.*
